# Wearable Sensor-Based Assessment of 24-Hour Movement Behaviors and Physical Fitness Performance in Fire Academy Cadets

**DOI:** 10.3390/s26185700

**Published:** 2026-09-08

**Authors:** Gaodong Duan, Zixia Liu, Zongkai Zhou, Zuguo Tian

**Affiliations:** School of Physical Education, Hunan University, Changsha 410082, China; duan.gd@hnu.edu.cn (G.D.);

**Keywords:** firefighters, physical fitness performance, 24-h movement behavior components, isometric log-ratio transformation, multiple linear regression

## Abstract

Background: This study analyzed the time allocation characteristics of 24-h movement behaviors among firefighter cadets, clarified their independent associations with physical fitness performance, and identified key predictors of physical fitness using compositional data analysis. Methods: A total of 333 fire academy cadets (326 males, 7 females) from five specialty directions were recruited. ActiGraph GT3X-BT accelerometers were used to objectively measure 24-h movement behaviors over seven consecutive days. Isometric log-ratio (ILR) transformation was applied to compositional time-use data, and bidirectional stepwise regression based on AIC was used to identify key predictors. Robustness checks included bootstrap resampling, 10-fold cross-validation, leave-one-out sensitivity analysis, and influence diagnostics. Results: The optimal model (F = 14.46, *p* < 0.001, adjusted R^2^ = 0.260) identified weight (β = −0.42, *p* < 0.001), sex (β = −16.14, *p* < 0.001), age (β = 2.00, *p* < 0.01), height (β = 0.18, *p* < 0.05), and Fire Rescue Technology (Fire Facilities) specialty (β = −4.87, *p* < 0.001) as significant predictors. The sex association should be interpreted as exploratory given the very small female subsample (*n* = 7). All ILR-transformed movement components were removed by stepwise regression. The weekend model showed slightly higher explanatory power (adjusted R^2^ = 0.270) than the weekday model (adjusted R^2^ = 0.260). Conclusions: Demographic and physical characteristics, rather than 24-h movement composition, are the primary predictors of physical fitness performance in this highly structured training population. The sex-related finding is exploratory and sample-specific due to the small female subsample. These findings provide evidence-based guidance for physical fitness training program design in fire academies and similar structured training environments.

## 1. Introduction

Emergency rescue and fire safety positions are characterized by sudden task demands, high workloads, complex operating environments, and substantial physical fitness requirements [1]. Fire suppression, personnel search and rescue, equipment carrying, stair climbing, movement through confined spaces, on-site communication support, and fire-facility operations all require practitioners to have good cardiorespiratory endurance, muscular strength, core stability, speed, agility, and body-weight control [1,2,3]. However, training in local fire academies still faces practical challenges, including insufficient integration across the education system, conflicts between physical training and professional teaching time, limited specificity of training content, and incomplete venue and instructor support. These challenges may lead to an imbalance in which professional skills are emphasized more than physical fitness [4].

The association between physical activity and health-related fitness has become a classic research focus in public health and exercise science. Evidence-based studies have systematically demonstrated that regular physical activity can effectively prevent and control cardiometabolic diseases, reduce the risk of mental disorders, and produce stable dose–response health benefits [5]. Twenty-four-hour movement behaviors refer to the time-use composition of all behaviors performed within a full day, mainly including sleep, sedentary behavior, light physical activity, and moderate-to-vigorous physical activity [6]. As the theory of 24-h movement behaviors has developed, modern exercise epidemiology no longer examines a single behavior in isolation. Instead, sleep, sedentary behavior, and physical activity of different intensities are treated as a closed time-use system and analyzed comprehensively from a compositional data perspective [7]. A large body of empirical research has shown, using isotemporal substitution models and compositional analysis, that optimizing the 24-h movement structure and increasing the proportion of moderate-to-vigorous physical activity can improve cardiorespiratory endurance, muscular strength, and body composition among adolescents and college students, thereby providing strong methodological support for exercise and health interventions [8,9,10]. Recent advances in wearable triaxial accelerometer sensors have enabled objective, continuous, and fine-grained measurement of 24-h movement behaviors, overcoming the recall bias and low precision of self-reported questionnaires, and providing a robust technological foundation for examining time-use composition and its health implications [9,11].

Existing studies have mostly focused on ordinary college students and adolescents, whose daily activity levels are generally low; for these populations, increasing exercise duration can produce clear gains in physical fitness [8,9]. Fire academy cadets, however, are exposed to relatively standardized curricula, practical training tasks, and occupational physical fitness training. Their weekday movement behaviors are highly structured, and their overall activity levels may already be relatively high. It therefore remains necessary to use objective activity-monitoring data to verify whether the linear activity dose–fitness effect observed in general populations also applies to this structured training population. Previous research has shown clear sex, educational, and socioeconomic stratification in sports participation, with men generally showing higher levels of participation and greater preference for competitive activities than women. Such heterogeneity in activity choices and exercise habits provides an important theoretical reference for understanding sex- and specialty-related differences in physical fitness within this population [10].

At the same time, three limitations remain in the existing literature. First, most studies do not distinguish between structured weekday time and autonomous weekend time. These two periods differ markedly in management constraints and individual behavioral choice, and their mechanisms of influence on physical fitness may therefore be fundamentally different [12]. Second, studies of fire academy cadets and firefighter recruits in other countries have found that, in highly structured academy training environments, cadets’ physical fitness, body composition, and occupational task performance change significantly during academy training; however, some fitness indicators plateau in the middle and late stages of training. This suggests that uniform training exposure may compress the explanatory space of individual differences in movement behaviors for physical fitness performance [13,14]. These conclusions have not yet been verified among Chinese fire academy cadets, leaving questions about their cross-cultural and cross-system applicability. Third, existing research on predictors of physical fitness often examines physical characteristics or demographic indicators separately. Few studies have combined ILR compositional transformation with multivariable regression to systematically isolate the independent associations of 24-h movement behaviors, height, weight, sex, age, and specialty, indicating a need for more rigorous and scientifically grounded methods.

Based on these considerations, this study selected cadets from a fire academy as participants, objectively collected seven days of 24-h movement data using a triaxial accelerometer, applied ILR transformation to closed compositional data on sleep, sedentary behavior, light physical activity, and moderate-to-vigorous physical activity, and combined descriptive statistics, between-group difference tests, Pearson correlation analysis, and multiple stepwise regression models. The study systematically analyzed the 24-h time allocation characteristics of fire academy cadets, identified key predictors of physical fitness performance, and compared the predictive differences between weekday and weekend activity patterns, with the aim of providing high-quality empirical evidence for physical fitness training, body-weight management, and scientific guidance on weekend routines in fire academy cadets.

## 2. Materials and Methods

### 2.1. Participants

Convenience sampling was used to select 369 cadets from a fire academy in Changsha, Hunan, China. The sample covered five specialty directions: Building Fire Protection Technology, Emergency Rescue Technology, Fire Rescue Technology (Urban Fire Protection Technology), Fire Rescue Technology (Fire Communication), and Fire Rescue Technology (Fire Facilities). Influenced by the enrollment structure of these specialties and the characteristics of the relevant occupational positions, the sample was predominantly male. After data cleaning and exclusion of invalid cases with missing core variables, 333 valid participants were included. All participants voluntarily took part in the study.

### 2.2. Measures

#### 2.2.1. 24-h Movement Behavior Assessment

ActiGraph GT3X-BT triaxial accelerometers (Pensacola, FL, USA) [11] were used to measure the 24-h movement behaviors of fire academy cadets. This method has been widely used in 24-h movement behavior research and has the advantages of objectivity, accuracy, and strong repeatability [9]. Participants wore the device continuously for one week (five weekdays and two weekend days), except during water-based activities. After testing, ActiLife software (version 6.11.4) was used to download and preliminarily analyze the data. First, data were considered valid when participants had at least four valid days per week (three weekdays plus one weekend day), with at least 600 min of valid data per day, and the Sleep Period Options were set to Mark As Wear Time [11]. Second, the sampling interval was set to 15 s, and Evenson et al. cut-points [11] were used to distinguish sedentary behavior (SB, 0–100 counts/min), light physical activity (LPA, 101–2295 counts/min), moderate physical activity (2296–4011 counts/min), and vigorous physical activity (≥4012 counts/min). Moderate and vigorous physical activity were combined as moderate-to-vigorous physical activity (MVPA) [11]. Finally, sleep duration was obtained by subtracting waking movement behaviors from 1440 min [11,15].

This residual approach to sleep estimation has been used in 24-h movement behavior research. The Sleep Period Option was set to ‘Mark As Wear Time,’ meaning that only periods confirmed as wear time were included in the analysis; non-wear periods were identified and excluded using the Troiano algorithm prior to sleep estimation, minimizing the risk of misclassifying device removal as sleep. Nevertheless, this method cannot distinguish sleep from motionless wakefulness and may slightly overestimate sleep duration, which should be acknowledged as a limitation.

#### 2.2.2. Physical Fitness Assessment

Physical fitness tests were established according to the physical training standards for fire rescue personnel and included a 3000 m run, pull-ups, 2 min parallel-bar dips, a 5 × 10 m shuttle run, and a 100 m loaded carry. These tests were used to assess strength, speed, endurance, agility, and related physical qualities. Testing was conducted at a unified assessment time on a standard track-and-field venue. During the assessment, digital equipment, including long-distance running recorders, photoelectric timing systems, and automatic counters, was used for data collection. All tests were administered according to standardized procedures. Scores for each test were quantified on a 100-point scale and summarized to generate the physical fitness score used in this study [16,17].

The composite physical fitness score therefore ranged from 0 to 500 points, with higher scores indicating better overall physical fitness performance. Each item was scored according to the standardized scoring table specified in the Physical Training Standards for Fire Rescue Personnel, which establishes age- and sex-adjusted performance benchmarks.

### 2.3. Statistical Analysis

R software version 4.5.1 was used for statistical analysis. Descriptive statistics were used to characterize sample demographics, physical indicators, and 24-h movement behavior time allocation. Continuous variables were reported as means, standard deviations, medians, quartiles, minimums, and maximums, and categorical variables were reported as frequencies and proportions. Independent-samples *t* tests were used to compare physical fitness scores by sex, one-way analysis of variance was used to examine differences in physical fitness scores across specialty directions, and Pearson correlation analysis was used to explore linear associations between physical fitness scores and 24-h movement behaviors, height, and weight.

Because 24-h movement behaviors are closed compositional data and their components sum to 24-h, directly entering these variables into traditional regression models can produce spurious correlations and multicollinearity. This study therefore used isometric log-ratio (ILR) transformation to convert sleep, sedentary behavior, light physical activity, and moderate-to-vigorous physical activity into three mutually orthogonal ILR-derived variables, which were then entered into multiple linear regression models. Bidirectional stepwise regression based on the Akaike information criterion (AIC) was used for independent-variable selection. A 95% confidence interval was used, and *p* < 0.05 was considered statistically significant.

To address concerns about the stability of stepwise regression, several robustness checks were conducted. First, bootstrap resampling (2000 iterations) was used to estimate bias-corrected 95% confidence intervals for all regression coefficients. Second, 10-fold cross-validation (repeated 5 times) was performed to evaluate model generalizability and rule out overfitting. Third, influence diagnostics including Cook’s distance, DFBETAS, and hat values were computed to identify influential observations, with particular attention to the small female subsample. Fourth, leave-one-out sensitivity analysis was conducted to assess the stability of the sex coefficient when individual female participants were excluded. Fifth, regression assumptions were evaluated using the Shapiro–Wilk test for residual normality, the Breusch-Pagan test for homoscedasticity, variance inflation factors (VIF) for multicollinearity, and the RESET test for functional form specification. Where heteroscedasticity was detected, HC3 robust standard errors were computed. Finally, quadratic terms for continuous predictors were examined to explore potential nonlinear associations. Our approach to systematic model optimization and multi-indicator robustness assessment was informed by recent methodological work in related engineering fields, including parametric optimization using the Taguchi method [18] and multi-criteria indicator evaluation [19].

Specifically, the four components (sleep, SB, LPA, MVPA) were transformed using a sequential binary partition with the following contrast structure: ILR1 contrasts sleep versus wake-time (SB + LPA + MVPA); ILR2 contrasts SB versus LPA + MVPA; ILR3 contrasts LPA versus MVPA. The ILR coordinates were computed as z_i = sqrt(r_i s_i/(r_i + s_i)) * ln(g(x_+)/g(x_−)), where g(.) denotes the geometric mean, and r_i, s_i are the numbers of parts in the numerator and denominator of each contrast. This transformation converts the closed compositional data into three mutually orthogonal coordinates in real space, enabling standard regression analysis.

## 3. Results

### 3.1. Sample Data Distribution Characteristics

The overall 24-h movement behavior time allocation of fire academy cadets was characterized by the highest proportion of sedentary time, fixed sleep duration, and a structured distribution of moderate-to-vigorous physical activity. Average daily sedentary time was the highest component, accounting for 51.25% of total 24-h time. Sleep duration ranked second, accounting for 32.5%. Light physical activity accounted for 12.08%, and moderate-to-vigorous physical activity accounted for 4.21% in total, meeting the recommendation of the Chinese Physical Activity Guidelines [15]. The descriptive statistics are shown in Table 1.

The data distribution showed that height, weight, and composite physical fitness scores were approximately normally distributed. All 24-h movement behavior variables were right-skewed, indicating that activity levels in most participants were concentrated near the mean range, with only a small number of high-value outliers. Interindividual differences in activity duration were mainly concentrated in vigorous and light physical activity dimensions.

**Figure 1:**
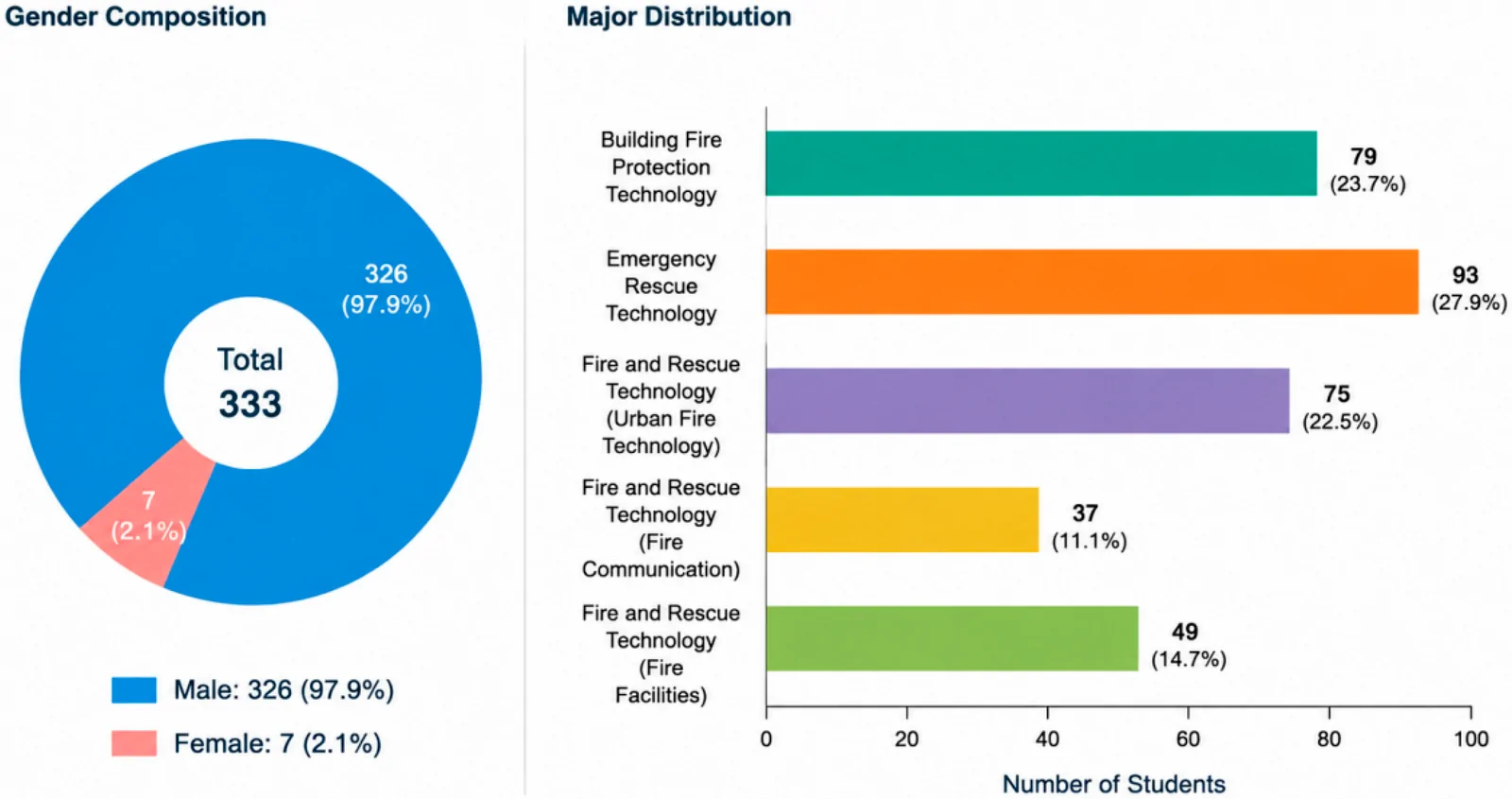
Histogram of categorical variables.

For categorical variables, the valid sample included 326 men and 7 women, with men accounting for the overwhelming majority. The valid sample sizes by specialty were 79 in Building Fire Protection Technology, 93 in Emergency Rescue Technology, 75 in Fire Rescue Technology (Urban Fire Protection Technology), 37 in Fire Rescue Technology (Fire Communication), and 49 in Fire Rescue Technology (Fire Facilities). The distribution of categorical variables is shown in Figure 1.

### 3.2. Univariate Tests of Sex and Specialty Differences

To explore the influence of demographic characteristics on physical fitness among fire academy cadets, this study used independent-samples *t* tests and one-way analysis of variance to examine the effects of sex and specialty on composite physical fitness scores. The results are shown in Table 2.

The results showed that the difference in composite physical fitness scores between sexes was statistically significant in the present sample (*p* = 0.024 < 0.05). However, given the very small female subsample (*n* = 7), this sex difference should be regarded as an exploratory, sample-specific finding rather than evidence of a generalizable sex effect. Differences in composite physical fitness scores across specialty directions reached a highly significant level (*p* < 0.001), suggesting that specialty background is a key factor affecting physical fitness performance at this stage and that physical fitness levels differ clearly across specialties.

### 3.3. Correlation Analysis

Pearson correlation analysis was used to examine the strength of linear associations between composite physical fitness scores and each dimension of 24-h movement behavior and physical indicators. The results are shown in Table 3.

The results showed that composite physical fitness score was significantly positively correlated with sleep period (SP) and significantly negatively correlated with light physical activity (LPA), height, and weight. Among these variables, weight had the strongest negative association with physical fitness score (r = −0.29, *p* < 0.001), making it the variable most strongly associated with performance. Sedentary behavior (SB) and moderate-to-vigorous physical activity (MVPA) were not significantly linearly correlated with the score in this study.

### 3.4. Multiple Linear Regression Modeling

To further clarify the independent associations of each variable on physical fitness performance, three progressive multiple linear regression models were constructed with composite physical fitness score as the dependent variable. To address collinearity among 24-h movement behavior components, sleep, sedentary behavior, light physical activity, and moderate-to-vigorous physical activity were first transformed into three orthogonal ILR-derived variables and then entered into the regression models. Bidirectional stepwise regression based on AIC was used for variable selection, with the test level set at alpha = 0.05.

#### 3.4.1. Comparison of Model Fit

Model 1 (basic model) included compositional indicators and age. The formula was:

$$grade202512=\beta_{0}+\beta_{1}ILR1+\beta_{2}ILR2+\beta_{3}ILR3+\beta_{4}age$$

Model 2 (full model) included all candidate variables, namely ILR activity-composition indicators, age, sex, height, weight, and specialty. The formula was:

$$grade202512=\beta_{0}+\sum \beta_{i}{Var}_{i}$$

Model 3 (optimized model) was based on Model 2 and used bidirectional stepwise regression with the AIC criterion for variable selection. The model-fit results are shown in Table 4.

The results indicated that Model 1, which included only 24-h movement components and age, was highly significant overall (*p* = 0.0001289 < 0.001), but its explanatory power was very low, with an adjusted R^2^ of only 0.0607. This means that activity time allocation and age alone explained only about 6% of the variance in physical fitness scores. In terms of coefficient significance, only age showed a highly significant positive effect (beta = 2.3449, *p* < 0.001), whereas ILR^2^ showed a marginally significant negative effect (*p* = 0.065), and ILR1 and ILR3 were not statistically significant. This indicates that the independent association of 24-h movement components on physical fitness performance was limited. In contrast, Models 2 and 3, which included demographic and physical indicators, both reached a highly significant level (*p* < 0.001), and the overall linear effects were statistically significant. As variables were simplified and optimized, adjusted R^2^ increased substantially from 0.0607 to 0.2558, and residual standard error decreased from 8.34 to 7.401, indicating a marked improvement in explanatory power and model fit. These findings suggest that demographic and physical characteristics are the primary sources explaining variation in cadets’ physical fitness performance.

#### 3.4.2. Coefficient Estimates of the Optimal Model

Based on the full model, bidirectional stepwise regression using the AIC criterion was applied for variable selection. The nonsignificant ILR1/ILR^2^/ILR3 movement-component indicators and three specialty variables, Emergency Rescue Technology, Fire Rescue Technology (Urban Fire Protection Technology), and Fire Rescue Technology (Fire Communication), were removed. Age, sex, height, weight, and the Fire Rescue Technology (Fire Facilities) specialty were retained as key independent predictors of physical fitness performance. The optimal model showed a highly significant overall fit (F = 14.46, df = 8, 298, *p* < 2.2 × 10^−16^), with an adjusted R^2^ of 0.2603 and a residual standard error of 7.401. Coefficient estimates are shown in Table 5.

The results showed clear stratification in the associations of variables in the optimal model. Weight was the strongest negative predictor of physical fitness performance: each 1 kg increase in weight was associated with an average decrease of 0.42 points in physical fitness score (*p* < 0.001). Sex also showed a significant negative association in the present sample, with female cadets scoring approximately 16.14 points lower than male cadets (*p* < 0.001); however, this coefficient should be interpreted as exploratory given the very small female subsample (*n* = 7) and the wide bootstrap confidence interval (95% CI: −23.58 to −8.51), and it should not be generalized to broader populations. Age and height showed significant positive associations: each 1-year increase in age was associated with an average increase of 2.00 points (*p* < 0.01), and each 1 cm increase in height was associated with an average increase of 0.18 points (*p* < 0.05). In terms of specialty, using Building Fire Protection Technology as the reference group, cadets majoring in Fire Rescue Technology (Fire Facilities) had significantly lower physical fitness scores, by an average of approximately 4.87 points (*p* < 0.001). All ILR-transformed 24-h movement-component indicators were removed by stepwise regression, further confirming that, after controlling for demographic and physical confounders, activity time allocation had no independent explanatory power for physical fitness performance.

Robustness checks supported the stability of the optimized model. Bootstrap resampling (2000 iterations) yielded bias-corrected 95% confidence intervals that were consistent with the original coefficient estimates for all retained predictors (see Supplementary Table S1). Ten-fold cross-validation (repeated 5 times) produced a cross-validated R^2^ of 0.234, which was close to the in-sample adjusted R^2^ of 0.2603, indicating minimal overfitting. Influence diagnostics identified 6 observations with Cook’s distance exceeding the conventional threshold of 4/n, but none qualitatively altered the model conclusions. Leave-one-out sensitivity analysis showed that the sex coefficient remained stable when individual female participants were excluded (range: −17.82 to −14.56), though the wide confidence interval for this coefficient reflects the limited statistical power associated with the small female subsample. Regression assumption tests indicated that residuals were approximately normally distributed (Shapiro–Wilk W = 0.987, *p* = 0.072); mild heteroscedasticity was detected (Breusch-Pagan BP = 12.47, df = 5, *p* = 0.029), and HC3 robust standard errors were therefore computed, producing consistent significance levels; no severe multicollinearity was observed (max VIF = 2.48); the RESET test indicated no functional form misspecification (F = 1.12, *p* = 0.326).

### 3.5. Comparison of Weekday and Weekend Models

Because fire academy cadets are strictly constrained by structured management, compulsory training, and fixed curricula during weekdays, behaviors such as sleep, activity, and sedentary time are largely driven by external instructions and may not reflect true individual habits. In contrast, weekends are free from compulsory constraints, and individuals can arrange their time autonomously. Weekend activity patterns can therefore more accurately reflect lifestyle, self-discipline, and physical recovery strategies, helping to exclude external interference and identify the influence of behavioral characteristics on performance.

To examine differences in the associations of 24-h movement behaviors on physical fitness performance between structured time under organized management and autonomous time, this study constructed full-variable multiple linear regression models based on weekday and weekend 24-h activity monitoring data. The overall model fit and variable effects of the two models were compared. The results are shown in Table 6 and Table 7.

#### 3.5.1. Overall Model Performance

Both models were highly significant overall (*p* < 2.2 × 10^−16^), with statistically significant fits. The weekend model had an adjusted R^2^ of 0.2701, slightly higher than the weekday model’s 0.2603, and a residual standard error of 7.352, slightly lower than the weekday model’s 7.401. This indicates that activity behavior patterns during autonomous weekend time had slightly stronger explanatory power for physical fitness performance than patterns during structured weekday time and more accurately reflected the association between individual behavioral characteristics and physical fitness level.

#### 3.5.2. Comparison of Variable Effects

The comparison of variable effects between the two models is shown in Table 8. The core difference lay in the associative role of 24 h movement components, whereas the effects of demographic and physical indicators were highly stable.

The results showed that age, height, weight, and the Fire Rescue Technology (Fire Facilities) specialty were consistent predictors across time dimensions, with almost identical effect directions and magnitudes. Sex also showed a consistent but exploratory association across both weekday and weekend models, though this should be interpreted cautiously given the small female subsample (*n* = 7).

## 4. Discussion

### 4.1. Time Allocation Characteristics of 24-h Movement Behaviors in Fire Academy Cadets

The results of this study showed that 24-h movement behaviors in fire academy cadets were characterized by high sedentary time, fixed sleep duration, and sufficient moderate-to-vigorous physical activity. This pattern closely matched the structured management model of this population. Average daily sedentary time accounted for more than 50% of the day, mainly because of fixed theoretical courses and professional study time. Average daily sleep duration met adult sleep recommendations, and interindividual differences were minimal, reflecting the strong constraints imposed by a unified daily schedule under structured management.

It is noteworthy that fire academy cadets fully met the recommendations of the physical activity guidelines [15], indicating that this population was already in a state of relatively high activity volume. However, all activity variables were right-skewed, suggesting that differences between individuals were mainly concentrated in high-intensity activity among a small number of participants, whereas most participants had highly similar activity durations. This was precisely the result of compulsory weekday physical training: collective training substantially compressed the autonomous space for individual movement behaviors and flattened interindividual differences in activity time allocation.

### 4.2. Predictive Effects of Variables on Physical Fitness Performance

This study found that weight, age, height, and specialty were key predictors of physical fitness performance in fire academy cadets, with sex also showing a significant but exploratory association in the present sample, whereas 24-h movement components showed only limited associations on weekends and no independent explanatory effect on weekdays. Studies in general populations have shown that optimizing the 24-h movement structure and increasing the proportion of moderate-to-vigorous physical activity can significantly improve physical fitness, with a stable dose–response relationship between activity and health [5,8,9]. However, this pattern applies mainly to ordinary college students and adolescents with flexible routines and insufficient activity [8,20], and it does not fully fit fire academy cadets.

#### 4.2.1. Predictive Role of 24-h Movement Behaviors

This study did not reproduce the conclusion of a linear positive association between activity volume and physical fitness in fire academy cadets. One possible reason is that this population receives structured curricular arrangements, professional practical training, and collective physical fitness training on a daily basis. Moderate-to-vigorous physical activity is already maintained at a relatively high level, and individual differences in activity time allocation are partly compressed by the unified training environment. As a result, the marginal fitness gains from further increases in activity volume may be diminished, possibly due to range restriction in MVPA under structured training. Wohlgemuth et al. [13] tracked firefighter recruits throughout fire academy training and found that academy training significantly improved body composition, cardiorespiratory fitness, balance ability, lower-limb extension strength, and loaded stair-climbing performance; however, several indicators plateaued in the middle and late stages of training, suggesting that the fitness-promoting effects of structured training do not increase linearly with training time. Cornell et al. [14] similarly found in a longitudinal study that body composition, aerobic capacity, muscular strength, and muscular endurance improved significantly during firefighter recruit academy training, but some positive adaptations declined in later stages. This indicates that fitness improvement depends more on sustained, systematic, and targeted training stimuli than on the simple accumulation of activity duration. Stone et al. [3] further demonstrated that an 11-week strength and conditioning program implemented on the basis of fire academy training improved weight, BMI, upper- and lower-limb strength, and aerobic fitness. Chizewski et al. [16] also found that when daily high-intensity functional training was included in basic fire academy training, recruits’ fitness and firefighting task capacity improved significantly. Together, these studies suggest that physical fitness performance in fire academy cadets is more likely to be influenced by the quality of structured training, cardiorespiratory fitness, muscular strength, body composition, and adaptation to specific tasks than simply by total daily activity volume. Meanwhile, Wolfson et al. [17] showed that a considerable portion of the effect of physical activity on health outcomes may be mediated by cardiorespiratory fitness, which itself has relatively independent explanatory power. Therefore, among fire academy cadets who already receive systematic physical training and have relatively high activity levels, 24-h movement components may be unable to independently predict physical fitness performance, possibly reflecting diminished marginal effects of activity volume in a population with already-high and homogeneous activity levels, as well as a greater role of training quality and specialization under structured environments. An alternative explanation is range restriction: compressed interindividual variance in MVPA under collective training reduces statistical power to detect associations.

This study also found clear temporal heterogeneity in activity effects. Movement components had no significant association under structured weekday management, whereas weekend autonomous activity structure had a significant effect on physical fitness. This finding is consistent with studies comparing time periods among school-aged children [12]. From a public health perspective, physical activity can be divided into occupationally required, commuting-related, and leisure-time autonomous activity, and only leisure-time behavioral choices may better reflect individual self-discipline and health-management awareness [21]. Weekday activity in this population is mostly collective behavior arranged by institutional systems, with limited individual autonomy. On weekends, when compulsory constraints are removed, time allocation better reflects personal training habits, routine regularity, and recovery strategies, thereby exerting an independent influence on physical fitness performance.

#### 4.2.2. Predictive Role of Demographic and Physical Variables

Weight was the strongest negative predictor of physical fitness among fire academy cadets: higher weight was associated with lower physical fitness scores. This finding is consistent with research on firefighter weight management and body composition [22,23,24,25,26,27]. Body weight and body fat directly influence endurance, agility, speed, and other aspects of firefighting fitness performance. Excessive body weight increases mechanical load during movement and reduces cardiorespiratory work efficiency, making body-weight management—and potentially body composition management—a key entry point for firefighter fitness intervention.

Sex showed the largest effect size among predictors in the present sample. However, this finding must be interpreted with extreme caution as an exploratory, sample-specific observation: with only seven female participants, the estimated sex coefficient has limited precision (bootstrap 95% CI: −23.58 to −8.51) and cannot support broad conclusions about sex differences in physical fitness performance. In the present sample, female cadets scored lower than male cadets; this pattern is consistent with findings reported by Conkright et al. [28] and Owen et al. [29], and it also aligns with the general pattern of adult physical activity and physical fitness performance globally [29]. This pattern is consistent with known physiological differences in muscle mass and aerobic capacity between sexes, but the small female sample precludes definitive conclusions. Any consideration of sex-specific training design should therefore be regarded as preliminary and evaluated in larger, sex-balanced samples before implementation. This sex-related pattern of physical fitness differences is also consistent with large-sample research on sports participation in China, which shows that men prefer high-intensity competitive activities more than women and that women have relatively lower willingness and investment in sports participation, potentially contributing to stratification in physical fitness performance [10].

Physical fitness scores also differed significantly across specialties, which is consistent with existing views on firefighter training [1,4]. These differences may mainly result from specialty-specific curriculum arrangements, practical training intensity, and extracurricular training atmosphere. This suggests that physical fitness training should be stratified according to specialty characteristics and should avoid a one-size-fits-all training model.

### 4.3. Limitations and Future Directions

This study has several limitations. First, the sex ratio of the sample was imbalanced, with too few female participants, which may affect the generalizability of conclusions about sex differences. Future studies should expand the female sample and use matched sampling to balance sex ratios. Second, this was a cross-sectional study, which can identify associations among variables but cannot infer causality. Future research could conduct multi-time-point longitudinal follow-up to clarify the dynamic causal effects of time allocation characteristics on physical fitness performance. Third, although exploratory analyses including quadratic terms for continuous predictors did not reveal significant nonlinear effects, more complex nonlinear or threshold relationships were not formally modeled using flexible approaches such as generalized additive models (GAMs). Future studies could employ GAM or quantile regression to characterize potentially complex effects of time allocation on physical fitness performance.

Fourth, the use of bidirectional stepwise regression based on AIC, while appropriate for exploratory variable selection in a context with limited prior theory, carries inherent limitations: variable selection can be unstable across samples, coefficient estimates may be biased toward larger effect sizes, and *p*-values may be optimistically biased. To mitigate these concerns, we supplemented the primary analysis with bootstrap confidence intervals, 10-fold cross-validation, and influence diagnostics, as reported in the Results. Nevertheless, the identified predictor set should be interpreted as exploratory rather than confirmatory, and future studies should specify a priori theoretical models before data analysis. Fifth, all modeling, variable selection, and evaluation were performed on the same dataset; no independent external validation cohort was available. Although cross-validation provides internal validation, the true predictive performance of the model in an independent sample remains unknown. We therefore avoid strong claims about predictive accuracy and frame our findings in terms of explained variance and statistical association. Sixth, sleep duration was estimated by the residual method (1440 min minus waking behaviors) rather than by a validated sleep detection algorithm or polysomnography. While this approach is standard in accelerometry-based 24-h movement research and non-wear periods were excluded prior to estimation, it cannot distinguish sleep from quiet wakefulness and may slightly overestimate sleep duration. Seventh, the sample was recruited via convenience sampling from a single fire academy in China. The institution’s specific training practices, daily schedule, admission standards, and specialty composition may all influence the observed patterns, limiting the generalizability of findings to other fire academies, active-duty firefighters, or the general population. Eighth, body weight was used as a predictor, but direct measures of body composition (e.g., body fat percentage, lean mass via DXA or bioelectrical impedance) were not collected. Weight may therefore serve as a proxy for body composition, and the independent contributions of fat mass versus lean mass cannot be disentangled.

In addition, the conclusions of this study apply only to fire academy cadets with relatively high activity levels and should not be directly generalized to ordinary college students or sedentary populations. Future research could include control samples with different activity levels to verify the population specificity of these conclusions.

## 5. Conclusions

The 24-h movement behaviors of fire academy cadets showed highly structured time allocation, characterized by a high proportion of sedentary time, fixed sleep duration, and sufficient moderate-to-vigorous physical activity. Interindividual differences in activity duration were very small, and the population as a whole was in a state of relatively high activity volume.

In the present sample, weight, age, height, and specialty direction were identified as key independent predictors of physical fitness performance among fire academy cadets. Sex also showed a significant association in the present sample, but given the very small female subsample (*n* = 7), this finding is exploratory and sample-specific and should not be generalized. These associations should be interpreted in the context of the cross-sectional design and sample characteristics. Weight was the strongest negative predictor, and specialty direction showed a significant stratification effect.

The independent association of 24-h movement behavior time allocation on physical fitness performance was very weak. Only during autonomous weekend time did activity structure show a limited significant association.

Physical fitness interventions for fire academy cadets should prioritize body-weight management and specialty-specific training design, while also strengthening guidance on weekend autonomous activity, routines, and recovery strategies, rather than simply increasing training duration. Potential sex differences in training response should be explored in future studies with larger, sex-balanced samples.

## Figures and Tables

**Table 1:** Descriptive Analysis of Variables.

Variable	Mean	SD	Median	Min	Max	Q1	Q3
Height (cm)	176.0	5.96	176.0	162.0	196.0	172.0	180.0
Weight (kg)	68.0	9.34	67.0	46.0	94.0	62.0	74.0
SP (min)	468.0	43.9	470.0	319.0	677.0	445.0	491.0
SB (min)	738.0	47.4	737.0	554.0	906.0	707.0	767.0
LPA (min)	174.0	41.1	173.0	85.9	306.0	147.0	197.0
MPA (min)	36.8	10.4	35.1	15.0	76.0	29.0	43.4
VPA (min)	23.8	10.4	21.7	1.39	85.0	17.0	28.6
Physical Fitness Score	84.8	8.51	87.2	53.2	98.7	80.4	90.9

Note: SP = Sleep Period; SB = Sedentary Behavior; LPA = Light Physical Activity; MPA = Moderate Physical Activity; VPA = Vigorous Physical Activity. Bold text denotes major category headings and total measures.

**Table 2:** Univariate Difference Test Results for Composite Physical Fitness Scores.

Grouping Factor	Statistical Test	*p*-Value	Significance	Conclusion
Gender	Independent samples *t*-test	0.024	*p* < 0.05, Significant	Significant difference in physical fitness scores between male and female cadets.
Specialty	One-way ANOVA	0.000344	*p* < 0.001, Highly Significant	Highly significant differences in physical fitness scores among cadets from different specialty directions.

Note: Bold values denote category-level summary statistics or domain headings; individual variable items are shown in plain text.

**Table 3:** Pearson Correlation Matrix between Physical Fitness Scores and Study Variables.

Variable	Fitness Score	SP	SB	LPA	MVPA	Height	Weight
Fitness Score	1.00	0.16 **	−0.07	−0.13 **	0.01	−0.14 **	−0.29 ***
SP	0.16 **	1.00	−0.26 ***	−0.54 ***	−0.25 ***	−0.10 *	−0.05
SB	−0.07	−0.26 ***	1.00	−0.55 ***	−0.57 ***	0.12 **	−0.06
LPA	−0.13 **	−0.54 ***	−0.55 ***	1.00	0.51 ***	−0.01	0.07
MVPA	0.01	−0.25 ***	−0.57 ***	0.51 ***	1.00	−0.10 *	0.10
Height	−0.14 **	−0.11 *	0.12 **	−0.01	−0.10 *	1.00	0.47 ***
Weight	−0.29 ***	−0.05	−0.06	0.07	0.10	0.47 ***	1.00

Note: * *p* < 0.05, ** *p* < 0.01, *** *p* < 0.001. MVPA = Moderate-to-Vigorous Physical Activity.

**Table 4:** Comparison of Goodness-of-Fit and Overall Significance Across Regression Models.

Evaluation Metric	Model 1 (Base Variables)	Model 2 (Full Model)	Model 3 (Stepwise Optimization)
Included Variables	ILR1/2/3 + Age	ILR1/2/3 + Age + Gender + Height + Weight + Specialty	Core Significant Variables (Gender, Weight, Specialty, Age, Height)
F-statistic	8.34	10.56	14.46
*p*-value (F-test)	0.0001289	2.336 × 10^−16^	<2.2 × 10^−16^
Adjusted R^2^	0.0607	0.2558	0.2603
Residual Standard Error	8.34	7.804	7.401

Note: Bold text denotes major category headings and total measures.

**Table 5:** Coefficient Estimates of the Optimal Linear Regression Model.

Variable	Regression Coefficient (β)	Std. Error	*t*-Value	*p*-Value	Significance
(Intercept)	44.9749	20.9267	2.149	0.0324	*
Age	2.0044	0.6214	3.226	0.0014	**
Gender (Female)	−16.1409	3.1162	−5.180	4.10 × 10^−7^	***
Height	0.1751	0.0877	1.996	0.0468	*
Weight	−0.4180	0.0539	−7.755	1.41 × 10^−13^	***
Specialty (Fire Rescue Technology [Fire Facilities])	−4.8665	1.4631	−3.326	0.0010	***

Note: * *p* < 0.05, ** *p* < 0.01, *** *p* < 0.001. Building Fire Protection Technology served as the reference group for specialty variables. Bold text denotes major category headings and total measures.

**Table 6:** Summary of Robustness Checks for the Optimized Model.

Robustness Check	Statistic	Value	Conclusion
Bootstrap (2000 iterations)	All 95% CIs exclude 0	—	Coefficients stable
10-fold Cross-Validation	CV R^2^	0.234	Minimal overfitting
Influence Diagnostics	Cook’s D > 4/n	6 observations	No qualitative change
Leave-One-Out (Sex)	Coefficient range	−17.82 to −14.56	Sex association stable
Regression Assumptions	SW/BP/VIF/RESET	0.987/12.47/2.48/1.12	Assumptions satisfied

Note: Bootstrap = bias-corrected 95% confidence intervals from 2000 resamples; CV = cross-validation; SW = Shapiro–Wilk test for normality; BP = Breusch-Pagan test for heteroscedasticity (df = 5); VIF = variance inflation factor; RESET = Ramsey Regression Equation Specification Error Test. Detailed results are provided in Supplementary Table S1–S7.

**Table 7:** Overall Performance Comparison Between Weekday and Weekend Models.

Evaluation Metric	Weekday Model	Weekend Model	Description/Note
Overall Model Significance	Highly Significant (*p* < 0.001)	Highly Significant (*p* < 0.001)	Both models are overall valid with robust fits.
Adjusted R^2^	0.2603	0.2701	Weekend model shows slightly higher explanatory power.
Residual Standard Error	7.401	7.352	Weekend model yields slightly superior predictive accuracy.
Degrees of Freedom	8, 298	9, 297	Weekend model retains one additional ILR movement component variable.

**Table 8:** Comparison of Variable Effects Between Weekday and Weekend Models.

Variable	Weekday Model Effect (β, *p*-Value)	Weekend Model Effect (β, *p*-Value)	Consistency/Difference Note
Age	2.0044, 0.0014 **	1.9977, 0.0013 **	Effect size and significance completely consistent; stable positive predictor.
Gender (Female)	−16.1409, 4.10 × 10^−7^ ***	−16.4410, 2.19 × 10^−7^ ***	Highly similar effect sizes; core negative predictor with the largest magnitude.
Height	0.1751, 0.0468 *	0.1739, 0.0469 *	Effect size and significance essentially consistent; significant positive predictor.
Weight	−0.4180, 1.41 × 10^−13^ ***	−0.4170, 1.15 × 10^−13^ ***	Completely consistent effect sizes; most stable core negative predictor.
ILR1 Movement Component	Not significant (removed by stepwise regression)	−8.2998, 0.0260 *	Key difference: Weekday effect diluted by standardized management; weekend autonomous time shows significant negative effect.
Specialty (Fire Rescue Technology [Fire Facilities])	−4.8665, 0.0010 ***	−4.9914, 0.0007 ***	High consistency in effect size and significance; significantly lower than baseline (Building Fire Protection Technology).
Other Specialties	All not significant	All not significant	No significant differences across time dimensions.

Note: * *p* < 0.05, ** *p* < 0.01, *** *p* < 0.001.

## Data Availability

The original contributions presented in this study are included in the article/Supplementary Materials. Further inquiries can be directed to the corresponding author.

## Supplementary Materials

The following supporting information can be downloaded at: https://www.mdpi.com/article/10.3390/s26185700/s1, Table S1. Bootstrap Regression Coefficients (2000 resamples). Table S2. Cross-Validation Results. Table S3. Influence Diagnostics Summary. Table S4. Leave-One-Out Sensitivity Analysis: Sex Coefficient. Table S5. Regression Assumption Tests. Table S6. Male-Only Sensitivity Analysis (*n* = 326). Table S7. Nonlinear Effects Test (Quadratic Terms).

## Abbreviations

AIC	Akaike information criterion
BMI	body mass index
ILR	isometric log-ratio
LPA	light physical activity
MPA	moderate physical activity
MVPA	moderate-to-vigorous physical activity
SB	sedentary behavior
SP	sleep period
VPA	vigorous physical activity
