# Supplementary material for: Wearable Sensor-Based Assessment of 24-Hour Movement Behaviors and Physical Fitness Performance in Fire Academy Cadets
Source: Sensors (Basel). 2026 Sep 8;26(18):5700. doi: 10.3390/s26185700 (PMC13611270; doi:10.3390/s26185700)
Supplement: Supplementary file 1 [file sensors-26-05700-s001.zip › sensors-4496543-supplementary.pdf]

Supplementary Materials

Table S1. Bootstrap Regression Coefficients (2000 resamples)

Bootstrap resampling with 2000 iterations was used to estimate bias-corrected 95% confidence intervals for all coefficients in the optimized multiple linear regression model (Model 3).

Variable	Original Estimate	Bootstrap Mean	Bootstrap SE	95% CI Lower	95% CI Upper
(Intercept)	42.35	42.18	8.92	24.76	59.74
Age	2.00	1.98	0.71	0.59	3.37
Sex (Female)	-16.14	-16.02	3.85	-23.58	-8.51
Height	0.18	0.17	0.08	0.02	0.33
Weight	-0.42	-0.41	0.10	-0.61	-0.22
Specialty: Fire Rescue Tech. (Fire Facilities)	-4.87	-4.82	1.42	-7.61	-2.08

Note: All bootstrap 95% confidence intervals exclude zero, consistent with the original model's significance levels. The sex coefficient shows the widest confidence interval, reflecting the limited statistical power associated with the small female subsample (n = 7).

Table S2. Cross-Validation Results

Ten-fold cross-validation (repeated 5 times) was performed to evaluate model generalizability and rule out overfitting.

Method	R^2	RMSE	MAE
In-sample (adjusted R^2)	0.260	7.40	5.82
10-fold CV	0.234	7.68	6.01
Repeated 10-fold CV (5 repeats)	0.231	7.71	6.04

Note: The cross-validated R^2 (0.234) is only slightly lower than the in-sample adjusted R^2 (0.260), indicating minimal overfitting. The difference of approximately 0.026 in R^2 is within expected range for a model with 8 predictors and n = 333.

Table S3. Influence Diagnostics Summary

Diagnostic	Threshold	Observations Exceeding Threshold	Impact on Conclusions
Cook's Distance	4/n = 0.012	6	None qualitatively altered model results
DFBETAS (Sex)	2/√n = 0.110	2	Sex coefficient remained negative and significant
Hat Values (Leverage)	2 × mean = 0.054	12	No single observation was disproportionately influential

Note: The 6 observations with elevated Cook's distance were primarily participants with extreme fitness scores or body weight values. Re-running the model excluding these observations produced consistent coefficient estimates and significance levels.

Table S4. Leave-One-Out Sensitivity Analysis: Sex Coefficient

The sex coefficient was re-estimated after sequentially excluding each of the 7 female participants.

Female Participant Excluded	Sex Coefficient	Change from Original (-16.14)
1	-16.82	-0.68
2	-15.47	+0.67
3	-17.82	-1.68
4	-14.56	+1.58
5	-16.31	-0.17
6	-15.89	+0.25
7	-16.58	-0.44
**Range**	**-17.82 to -14.56**	**±1.68**

Note: The sex coefficient remained negative and statistically significant ($p < 0.05$) across all leave-one-out iterations. The range of variation (±1.68) is relatively small compared to the original estimate (-16.14), suggesting the sex association is not driven

by any single female participant. However, the wide confidence interval (see Table S1) indicates limited precision in estimating this effect.

Table S5. Regression Assumption Tests

Assumption	Test	Statistic	p-value	Conclusion
Residual Normality	Shapiro-Wilk	W = 0.987	0.072	Approximately normal
Homoscedasticity	Breusch-Pagan	BP = 12.47 (df=5)	0.027	Mild heteroscedasticity detected
Multicollinearity	Max VIF	2.48	—	No severe multicollinearity
Functional Form	RESET	F = 1.12	0.326	No misspecification

Note: Mild heteroscedasticity was detected (p = 0.029). HC3 robust standard errors were computed as a sensitivity check and produced consistent significance levels for all predictors. The maximum VIF of 2.48 is well below the conventional threshold of 10, indicating no multicollinearity concern.

Table S6. Male-Only Sensitivity Analysis (n = 326)

The primary regression model was re-run excluding all female participants to verify that core findings do not depend on the small female subsample.

Variable	Full Sample (n=333)	Male Only (n=326)
Age	2.00 **	1.92 **
Height	0.18 *	0.19 *
Weight	-0.42 ***	-0.40 ***
Specialty: Fire Facilities	-4.87 ***	-4.71 ***
Adjusted R^2	0.260	0.248

Note: p < 0.05, **p < 0.01,** *p < 0.001. All core predictors remained statistically significant in the male-only subsample, with consistent effect directions and similar magnitudes. This confirms that the primary findings are not driven by the female subsample.*

Table S7. Nonlinear Effects Test (Quadratic Terms)

Quadratic terms were added for all continuous predictors (age, height, weight) to explore potential nonlinear associations.

Variable	Linear Coefficient	Quadratic Coefficient	p-value (Quadratic)
Age	2.15	-0.08	0.312
Height	0.21	-0.001	0.547
Weight	-0.45	0.002	0.289

Note: None of the quadratic terms reached statistical significance (all $p > 0.05$), indicating that linear specifications are adequate for the present data. The model with quadratic terms did not significantly improve fit compared to the linear model (ANOVA $F = 0.87$, $p = 0.458$).
